# Implantable Peripheral Nerve Stimulation for Peripheral Neuropathic Pain: A Systematic Review of Prospective Studies

**DOI:** 10.3390/biomedicines10102606

**Published:** 2022-10-17

**Authors:** Steven Char, Max Y. Jin, Vinicius Tieppo Francio, Nasir Hussain, Eric J. Wang, Mahmoud Morsi, Vwaire Orhurhu, Larry J. Prokop, Adam Fink, Ryan S. D’Souza

**Affiliations:** 1 Department of Anesthesiology & Perioperative Medicine, Rutgers New Jersey Medical School, Newark, NJ 07103, USA; 2 Department of Anesthesiology, University of Wisconsin-Madison, Madison, WI 53706, USA; 3 Department of Rehabilitation Medicine, The University of Kansas Medical Center, Kansas City, KS 66160, USA; 4 Department of Anesthesiology, The Ohio State Wexner Medical Center, Columbus, OH 43210, USA; 5 Department of Anesthesiology and Critical Care, Johns Hopkins University School of Medicine, Baltimore, MD 21218, USA; 6 Department of Anesthesiology & Pain Management, John H. Stroger, Jr. Hospital of Cook County, Chicago, IL 60612, USA; 7 Department of Anesthesiology, University of Pittsburgh Medical Center, Susquehanna, Williamsport, PA 17701, USA; 8 MVN Health, East Stroudsburg, PA 18301, USA; 9 Mayo Clinic Libraries, Mayo Clinic, Rochester, MN 55902, USA; 10 1st Faculty of Medicine, Charles University, 110 00 Prague, Czech Republic; 11 Department of Anesthesiology and Perioperative Medicine, Mayo Clinic Hospital, Rochester, MN 55902, USA

**Keywords:** peripheral nerve stimulator, peripheral neuropathy, chronic pain, neuromodulation

## Abstract

Peripheral nerve stimulation (PNS) has been utilized for over 50 years with accumulating evidence of efficacy in a variety of chronic pain conditions. The level and strength of evidence supporting the use of PNS for peripheral neuropathic pain remains unclear. The purpose of this review is to synthesize data from prospective studies on the efficacy of PNS for neuropathic pain as it pertains to pain intensity, neurological deficits/neuropathy (e.g., weakness, sensory deficits, gait/balance), and other secondary outcomes (quality of life, satisfaction, emotional functioning, and adverse events). In compliance with the Preferred Reporting Items for Systematic Reviews and Meta-analyses (PRISMA) guidelines, this review identified articles from MEDLINE(R), EMBASE, Cochrane Central Register of Controlled Trials, Cochrane Database of Systematic Reviews, and Scopus. Overall, per the Grading of Recommendations Assessment, Development and Evaluation (GRADE) criteria, pooled results demonstrate very low quality or low quality of evidence supporting modest to substantial improvement in pain and neurological function after PNS implantation for treatment of peripheral neuropathic pain. PNS for phantom limb pain was the only indication that had moderate level evidence. Future prospective and well-powered studies are warranted to assess the efficacy of PNS for peripheral neuropathic pain.

## 1. Introduction

The utilization of electrical stimulation of peripheral nerves has been documented for over 50 years. It was first described in the treatment of trigeminal neuralgia, and later utilized for chronic cutaneous neuropathic limb pain [1,2]. A decade later, the first clinical studies of implantable peripheral nerve stimulation (PNS) were performed, revealing pain reduction, increased function, and quality of life with use [3,4]. Unfortunately, the early adoption of PNS was poor due to early studies demonstrating modest effectiveness with the potential for concerning complications [5,6]. With surgical advancements in nerve dissection and visualization, techniques then evolved to allow for open lead implantation adjacent to peripheral nerves. However, it was two decades ago that the concept of percutaneous PNS lead placement was introduced [4,7]. Since then, visualization with fluoroscopy or ultrasound guidance, and percutaneous placement of miniaturized leads with external battery sources has facilitated a safer and minimally invasive placement of PNS devices. 

Currently, PNS is approved by the United States Food and Drug Administration (FDA) for the treatment of acute or chronic pain located in the low back, upper or lower extremities, and head [6,8,9]. Thus far, PNS has been applied for a variety conditions including: mononeuropathies, neuropathic limb pain, post-stroke shoulder pain, headache, plexus injuries, post-amputation pain or phantom limb pain (PLP), pelvic pain, complex regional pain syndrome (CRPS), and chronic low back pain.

Other neuromodulation modalities used in practice for the management of peripheral neuropathic pain include dorsal column spinal cord stimulation (SCS) and dorsal root ganglion stimulation (DRG-S). The literature has shown remarkable success rates with SCS and DRG-S [10,11]. In patients who experience failure of SCS therapy for CRPS, salvage of relief maybe attained with use of DRG-S [12]. 

Numerous mechanisms of action have been proposed to explain the therapeutic effects of PNS, including both peripheral and centrally acting mechanisms [8,10,11,13]. Central mechanisms may involve decrease of central sensitization and hyperalgesia by inhibiting dorsal horn interneuron activity and wide dynamic neuron activity, as well as modulating serotonergic and GABAergic biochemical pathways [6,10]. Peripherally, PNS may provide pain relief by selectively modulating the large diameter Aβ afferent nerve fibers without small fiber activation, thereby directly hindering transmission of pain impulses [10,13].

Despite the emergence of PNS treatment for a variety of painful conditions, the level and strength of evidence to support its use for peripheral neuropathic pain remains unclear. The main objective of this systematic review is to synthesize data on the effectiveness of PNS for neuropathic pain as it pertains to pain intensity (primary outcome), and secondary outcomes including neurological deficits/neuropathy (e.g., weakness, sensory deficits, gait/balance), quality of life, satisfaction, emotional functioning, and adverse events.

## 2. Materials and Methods

### 2.1. Search Strategy

This review abided to the Preferred Reporting Items for Systematic Reviews and Meta-Analysis (PRISMA) guidelines and had its protocol registered in the International Prospective Register of Systematic Reviews (PROSPERO ID CRD42022345599). A systematic search strategy was created in the English language for several databases from database inception to 5 July 2022. The databases included MEDLINE(R), EMBASE, Cochrane Central Register of Controlled Trials, Cochrane Database of Systematic Reviews, and Scopus. Syntax utilized in the search strategy included terms and synonyms for *peripheral nerve stimulation, peripheral neuropathy, peripheral neuropathic pain, complex regional pain syndrome, amputation pain, phantom limb pain*, and *brachial plexus*. The complete search syntax can be found in Supplementary Tables S1–S3.

### 2.2. Study Selection

The inclusion criteria consisted of any prospective human study design that utilized PNS to treat peripheral neuropathic pain. We considered only those studies that placed an implantable permanent or temporary PNS device. Further, we only included those studies that evaluated the use of PNS for upper or lower extremity pain; all other uses of the PNS device were excluded. We also excluded studies of animal models and those investigating the use of peripheral field stimulation. Two authors (S.C. and M.Y.J.) independently selected articles while a third author (R.S.D.) settled any disagreements.

### 2.3. Data Extraction

The following data was extracted from each included article: general study characteristics (study design, funding, number of participants, mean age of participants) and intervention data (waveform, stimulation settings, stimulation type) and all presented outcome data with timeframes of assessment. The primary outcome of interest for this review was the change in pain intensity related to peripheral neuropathic pain after PNS implant. Secondary outcomes of interest included changes in neurological function, changes in quality of life, emotional functioning, and adverse events. Two authors (M.M. and M.Y.J.) independently extracted data, while a third author (S.C.) resolved any discrepancies.

### 2.4. Assessment of Risk of Bias

Risk of bias was assessed using either the Newcastle–Ottawa Quality Assessment Scale (NOS) or the Cochrane Risk of Bias Tool (C-ROB). Observational studies were assessed using the NOS while the assessment of randomized controlled trials (RCTs) was completed using the C-ROB. For the NOS, studies were evaluated based on selection (representativeness of the exposed cohort, selection of the non-exposed cohort, ascertainment of exposure, demonstration that outcome of interest was not present at the start), comparability (comparability of cohorts on the basis of the design or analysis), and exposure/outcome (assessment of outcome, follow-up long enough for outcomes to occur, adequacy of follow-up of cohorts). A maximum of four stars can be obtained for the selection domain, while a maximum of two and three stars can be obtained for the comparability and exposure/outcome domains respectively. For each domain, a greater number of stars obtained indicates a lower risk of bias. The C-ROB assesses studies for bias based on the following domains: Selection, Performance, Detection, Attrition, Reporting, and other biases. Each domain could receive a score of high risk, low risk, or unclear risk. All bias assessments were independently completed by two authors (S.C. and M.M.) with a third author adjudicating any discrepancies (R.S.D.).

### 2.5. Quality Assessment

The Grading of Recommendations Assessment, Development, and Evaluation (GRADE) approach was used to assess the overall quality of evidence of PNS for treatment of pain intensity for each type of neuropathic pain (primary outcome only). The GRADE assessment uses standard criteria to evaluate the certainty of evidence as being of very low, low, moderate, and high. 

## 3. Results

**Figure 1:**
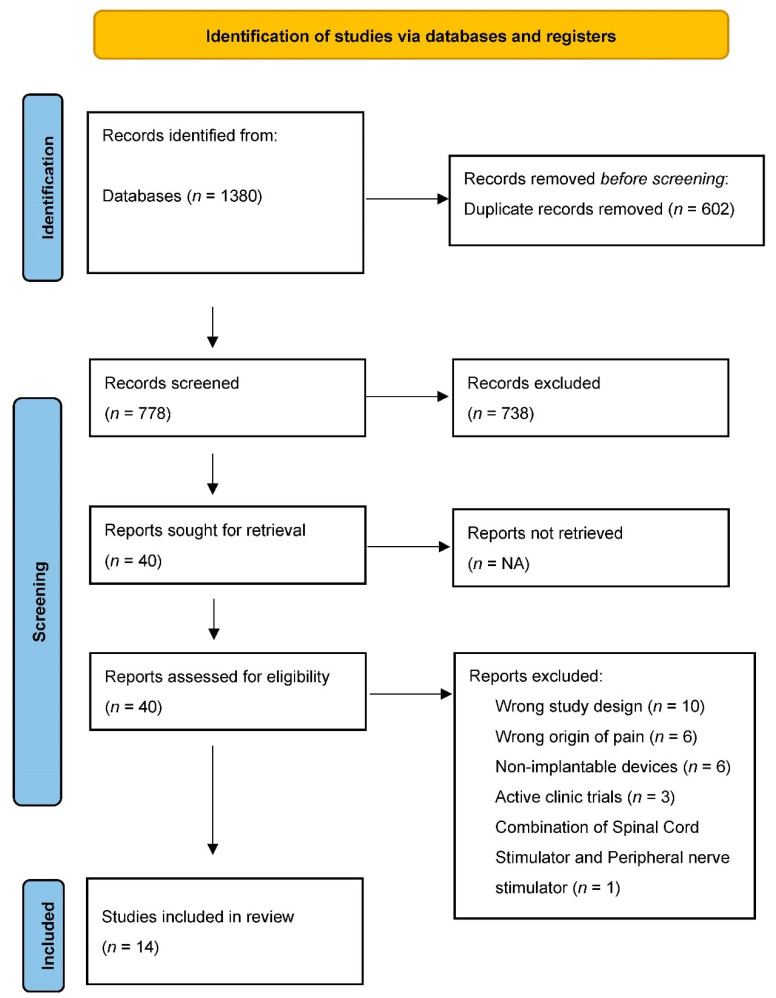
PRISMA Flow Chart. PRISMA flow diagram for systematic review. Flowchart of the study selection process, inclusion and exclusion of studies, and reasons for exclusion are displayed.

Our search results yielded 1380 citations. After duplicate screening, 778 citations had their title and abstracts screened for eligibility. After the initial screen based on title and abstract alone, 40 full-text articles were retrieved and assessed for their eligibility. We included 14 studies and their characteristics are shown in Table 1. Figure 1 (PRISMA) shows the results of the search and reasons for exclusion. Eleven studies were prospective observational studies/case series [14,15,16,17,18,19,20,21,22,23,24] while three [25,26,27] were randomized controlled trials (RCTs).

### 3.1. Type of Neuropathic Pain

#### 3.1.1. Complex Regional Pain Syndrome (CRPS) 

Complex Regional Pain Syndrome (CRPS) is defined as a painful condition that is disproportionate in time or degree to the usual course of any known trauma or other lesion. The pain is regional (not in a specific nerve territory or dermatome) and usually has a distal predominance of abnormal sensory, motor, sudomotor, vasomotor, and/or trophic findings^1^. Three studies [14,18,24] evaluated the use of PNS in patients diagnosed with treatment-resistant CRPS. Frederico et al. [14] included seven patients with CRPS I and three patients with CRPS II. At 12-month follow-up after PNS implantation, visual analog scale (VAS) score, Neuropathic Pain Scale (NPS), and Short Form-12 (SF-12) physical and mental component scores were analyzed. VAS, NPS and SF-12 improved by 57.4% ± 10% (*p* = 0.005), 60.2% ± 12.9% (*p* = 0.006), and 21.9% ± 5.9% (*p* = 0.015), respectively. Eight of the 10 patients showed a pain reduction > 50% on the VAS scale whereas the remaining two had a >30% reduction in pain intensity. No adverse events were reported. In a prospective clinical trial [18] involving six patients with CRPS, PNS of the tibial nerve was performed. From a baseline VAS score of 7.5, follow-up data revealed reduced VAS scores after 1 month (2.6, *p* = 0.03), 3 months (1.6, *p* = 0.03), and 6 months (1.3, *p* = 0.02). Secondary endpoints of the average McGill score before surgery was 23.8, 11.0 (*p* = 0.45) after 1 month, 6.3 (*p* = 0.043) after 3 months, and 4.5 (*p* = 0.01) after 6 months. Only 1–2 h of active stimulation with 10 to 20 Hz was sufficient and provided analgesia lasting 24 h in this cohort. Lastly, 30 patients with CRPS (median nerve affected, 7 patients; ulnar nerve, 10 patients; radial nerve, one patient; common peroneal nerve, five patients; and posterior tibial nerve, seven patients) underwent PNS implantation to the affected nerve [24]. The authors reported a reduction in pain intensity from 8.3 ± 0.3 preimplantation to 3.5 ± 0.4 (56.7% ± 5.0% reduction) at the latest follow up (*p* < 0.001). Furthermore, there was an increased level of activity by 63.3% ± 21.8% with four patients increasing employment from unemployed to full-time employment, two from unemployed to part-time employment, and two from part-time to full-time employment.

#### 3.1.2. Shoulder Pain

Implantation of PNS in patients with chronic shoulder pain was evaluated in two studies [16,20]. In a multi-site case series [16], five patients with poststroke shoulder pain received PNS to the axillary nerve. Using the Brief Pain Inventory Short Form (BPI-SF3), there was a reduction in pain intensity by 69.2% at 6 months (95% CI [1.8–5.5], *p* = 0.003), 84.6% at 12 months (95% CI [2.6–6.3], *p* = 0.0002), and 69.2% at 24 months (95% CI [1.7–5.5], *p* = 0.003) compared to prior device placement. All five participants experienced a 50% or greater pain reduction at 6 and 12 months after PNS, and four experienced at least a 50% reduction at 24 months after PNS. In a single-center, unblinded case series [20], PNS of the terminal branches of the axillary nerve in ten patients with chronic shoulder pain due to subacromial impingement syndrome was performed. Seven patients completed all outcome assessments with the primary outcome measure being BPI-SF3. There was a significant reduction in pain (BPI-SF3, *F*(1, 66) = 12.9, *p* < 0.01). After 16 weeks following implantation, average pain intensity among subjects was 8.2 (±standard error [SE] 1.1) and 4.2 (±SE 1.1). Apart from benign granuloma formation seen in seven patients, the authors reported no adverse events. 

#### 3.1.3. Phantom Limb Pain (PLP)

Phantom limb pain is defined as the perception of pain in the amputated portion of the limb after amputation. On the other hand, residual limb pain (RLP) is pain originating from the part of the limb that remains after an amputation. Three studies [22,25,26] evaluated PNS in patients with PLP. The first was a randomized, placebo-controlled, double-blind trial [26]. The primary outcome of treatment responders was defined as a ≥50% reduction in average daily pain score during weeks 1–4 of the treatment period in their RLP and PLP. The primary safety outcome was the occurrence of device-related and procedure-related adverse events assessed at all follow-up visits. Nine participants in the treatment group and six in the placebo group completed the 12-month follow-up period. At 12 months, 67% (6/9, *p* = 0.001) of participants receiving PNS treatment had sustained reductions of ≥50% in average pain in RLP and PLP over the week prior to the 12-month visit. No participants in the placebo group (0%, 0/14) reported ≥50% reductions in average weekly pain at the end of the placebo period. After crossing over to receive 4 weeks of active stimulation, the placebo group did report significant improvement in average PLP (33% reduction from baseline, *p* = 0.027) compared with placebo treatment during weeks 1–4. There were no serious or unanticipated study-related adverse events. 

The second study [27] was a multicenter, randomized, double-blind, placebo-controlled, partial-crossover study of 26 patients with 12-month follow-up data on PNS for postamputation pain. The femoral and sciatic nerves were stimulated. Overall, 7/12 (58%) patients receiving PNS reported >50% pain relief compared to 2/14 (14%) in the placebo group during weeks 1–4 of therapy. Among the seven patients who responded to therapy, the average reduction in RLP and PLP were 73% and 69%, respectively. In week 5–8 of therapy, 8/12 (67%) of patients receiving PNS reported >50% pain relief compared to 2/14 (14%) in placebo group. Notably, after crossing over at week 4, patients in the placebo group reported only significant improvement in PLP but not RLP. Furthermore, subjects reporting >50% pain improvement remained at 2/14 (14%). Lastly, Rauck et al. [22] enrolled 16 patients suffering from PLP. Nine of the 16 patients underwent PNS implantation. Among those who received PNS, reductions in mean daily worst post-amputation pain intensity (56 ± 26%, 56 ± 26%, *n* = 9), average RLP (72 ± 28%, 42 ± 27%, *n* = 7), average PLP (81 ± 28%, 47 ± 48%, *n* = 7), RLP interference (81 ± 27%, 53 ± 17%, *n* = 6), PLP interference (83 ± 31%, 56 ± 46%, *n* = 7), and Pain Disability Index (70 ± 38%, 55 ± 32%, *n* = 9) were observed during the second week of stimulation and four weeks after the end of stimulation, respectively.

#### 3.1.4. Post-Surgical Pain

Two studies [19,27] evaluated the effectiveness of PNS in patients with post-surgical pain. The first study [19] involved 29 patients with neuropathic, chronic postherniorrhaphy groin pain. Twenty-one patients (72.4%) presented with pain consistent with ilioinguinal nerve involvement. A total of seven patients received PNS implantation. After 3 months of follow-up, a significant reduction in pain from 8/10 to 2/10 on the NRS scale was observed (*p* < 0.001). Only one patient failed PNS therapy. 

The second study [27] was a prospective multicenter, randomized, double-blind, partial crossover, three stage group (upper extremities, lower extremities, trunk) sequential study. The primary outcomes were pain relief and adverse events. Pain relief was measured by average pain at rest using a numerical rating scale (NRS) at three months. Safety was determined by assessment of adverse events during the one-year study period. Ninety-four patients with chronic, intractable posttraumatic/postsurgical pain were implanted and then randomized to the treatment (*n* = 45) or the control group (*n* = 49). The primary effectiveness endpoint (≥30% decrease in the NRS pain score without any upward titration of the patient’s pain medicine regimen), three months after randomization to treatment, demonstrated that patients receiving active stimulation achieved a statistically significantly higher response rate of 38% vs. the 10% rate found in the control group (*p* = 0.0048). The overall mean pain reduction from baseline to three-month follow-up was 27.2% in the treatment group vs. 2.3% in the control group (*p* < 0.0001). Of the 94 subjects included in the study, 15 subjects did not participate in the 6- and 12-month follow-up, and an additional 33 did not follow up at the 12-month visit, representing an attrition rate of 51% (48/94). The authors reported no serious adverse events related with the device, but did note 14 adverse events in the treatment cohort and 13 adverse events in the control cohort. These events typically occurred and resolved early within the first three months of the study, and were largely localized to the stimulation area or site of surgery and were superficial in nature (e.g., skin rash, redness, soreness).

#### 3.1.5. Mononeuropathy

Five studies [15,17,21,23] used PNS therapy for focal mononeuropathies. One case series [15] of 39 patients used PNS for focal mononeuropathies where several different nerves were targeted, with the axillary nerve (*n* = 18 patients) being the most frequent. The average percent reduction of VAS pain scores ranged from 29 to 100%, and the magnitude of effectiveness varied by the nerve stimulated. Notably, all three patients who received PNS of the lateral femoral cutaneous nerve experienced a 100% change in VAS from 8.3 prior to implant to 0 after implant. The effect on activity was also noted to improve by 72% in all patients. Moreover, 89% of those implanted with a PNS observed a greater than 50% reduction in opioid consumption. In a prospective case series [17], 23 patients with painful mononeuropathy secondary to leprosy underwent PNS implantation. Follow up visits were conducted at 1, 3, 6 and 12 months after PNS implant. After a seven-day trial, it was found that 10 patients reported a >50% pain reduction on the VAS scale and the neuropathic pain scale. After 12 months, 6/10 had a pain reduction of >50% or greater. Seven patients with intractable post-traumatic brachial plexus lesions [21] received a quadripolar electrode lead placed directly on the sensory peripheral branch of the main nerve involved, proximal to the site of lesion, into the axillary cavity. The mean baseline NRS was 9/10, indicating moderate to severe pain intensity before surgery. Pain intensity decreased from an NRS of 9  ±  1.15 before surgery to 2.14  ±  1.57 at the 6-month follow-up and to 2.57  ±  1.13 at the 12-month follow-up (*p*  <  0.001). Lastly, eight patients with treatment-resistant carpal tunnel syndrome (CTS) [23] had PNS to stimulate the median nerve. The primary endpoint was pain relief near the median nerve and device safety. Overall, 2/10 patients (20%) experienced a >30% decrease in pain. Mean average pain scores were reduced from 6.7 pre-implant to 6.2 post-implant. In addition, 9/10 (90%) experienced 17–100% reduction in pain intensity on day 5 (at the end of stimulation) versus baseline, with an average pain reduction of 44.2%. After explant, pain scores returned to baseline, increasing 36.8% to 45.6% relative to the average reduced pain scores with daily stimulation. Three adverse events were reported, all of which were mild, unrelated to the device, and resolved uneventfully. 

#### 3.1.6. Bias Assessment

**Figure 2:**
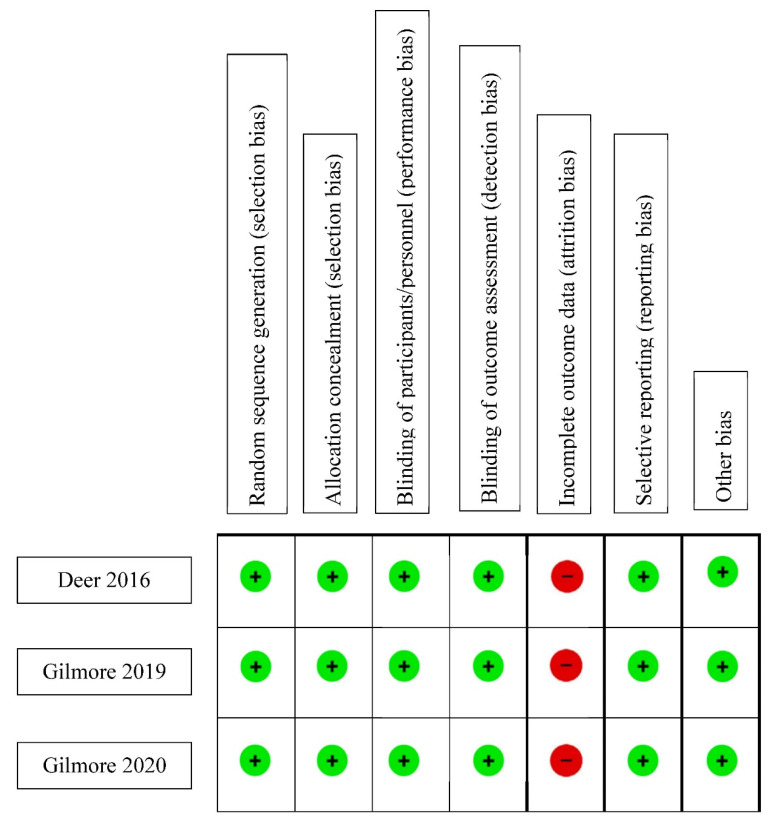
Cochrane Risk-of-Bias Assessment of prospective trials.

The risk of bias assessment of cohort studies is summarized in Table 2. An adequate follow-up period was determined to be at least six months, and 95% of total participants remaining under observation at the primary endpoint of the study was deemed adequate (e.g., <5% patients who dropped out). As none of the studies selected controls, a maximum of three stars could be awarded when evaluating for selection bias via the NOS due to an absence of a non-exposed cohort. No study was evaluated for comparability due to the same reason. With the exception of 4 studies [15,18,22,24], all began with a trial phase, after which only patients who met pre-determined response criteria progressed to the more permanent form of PNS. As such, all calculations regarding the duration of follow-up and percentage lost to follow-up were derived from the time and number of patients who entered the second phase of the respective studies. Apart from the absence of a control group, all studies demonstrated a low risk for selection bias. Five of the studies demonstrated moderate bias risk pertaining to outcomes, owing to a high percentage of patients lost to follow-up [15,17,23,24,27]. Figure 2 shows the C-ROB assessment of the three included RCTs [25,26,27]. All three studies demonstrated low risk for bias in all domains except attrition bias due to an attrition rate of 57% [25], 58% [26], and 57% [27]. 

#### 3.1.7. Quality of Evidence

Assessment using GRADE found that there was overall low-quality evidence supporting reduced pain intensity of peripheral neuropathic pain after treatment with PNS. While all included studies were prospective in nature, only three were RCTs, thus reducing the quality of evidence. Stratifying the GRADE quality of evidence assessment by type of peripheral neuropathic pain, low-quality evidence supported reduced pain intensity with PNS treatment for CRPS, shoulder pain, post-surgical pain, and mononeuropathies, and moderate-quality evidence for PLP. A summary table with the GRADE assessment is displayed in Table 3.

## 4. Discussion

This systematic review appraised evidence on changes in pain intensity in peripheral neuropathic pain after treatment with implantable PNS systems. Specific peripheral neuropathic pain syndromes included were CRPS, shoulder pain, PLP, post-surgical pain, and mononeuropathies of the extremities. PNS for peripheral neuropathic pain is a well-researched area of neuromodulation, with a plethora of available literature. As such, we aimed to only assess prospective studies and exclude all retrospective data. Across 14 prospective studies and one 12-month follow-up analysis of those prospective studies, overall findings suggest that there is low-quality evidence supporting that PNS has the ability to provide clinically meaningful pain relief for peripheral neuropathic pain. The majority of patients experienced at least a 30% reduction in pain, although it was common for patients to report greater than 50% pain relief. This reduction in pain was consistent across all types of peripheral neuropathic pain syndromes. These findings align with results from recent reviews of PNS treatment in chemotherapy-induced peripheral neuropathy [28] and other peripheral neuropathies [6,28,29,30,31].

SCS and DRG-S are also being utilized for managing patients with peripheral neuropathic pain. The literature has shown that in cases of appropriate patient selection, SCS can achieve a success rate in the range of 50–100%, approaching and even surpassing that of PNS [8,32]. However, the available evidence for DRG-S therapy for painful diabetic neuropathy (PDN) and polyneuropathy highlights low-quality GRADE evidence in pain reduction [9]. On the contrary, in the management of CRPS, a randomized, prospective trial showed clinical and statistical significance in pain relief, postural stability and mood improvements favoring DRG-S versus SCS therapy [32]. 

The exact mechanism by which PNS modulates peripheral neuropathic pain remains a subject of further inquiry and investigation. The hypotheses detailed in current literature can largely be divided into peripherally and centrally acting mechanisms [33]. The Gate Control Theory [34], originally proposed in 1965, hypothesized that the stimulation of large, myelinated, sensory nerve fibers exerts an inhibitory effect on the transmission of nociceptive information from smaller nerve fibers via the activation of dorsal horn interneurons. The Gate Control Theory remains the underlying foundation for the hypotheses attempting to explain the peripherally acting mechanism of PNS and has been demonstrated in both human [35] and animal [36] studies. Further research has suggested that PNS induces lower concentrations of neurotransmitters and local proinflammatory molecules in the peripheral nervous system, and that this effect plays a role in the modulation and attenuation of pain [37].

Centrally acting mechanisms also include alterations of neurotransmitter levels in the central nervous system, specifically in the serotonergic, glycinergic, and GABAergic pathways [33]. An additional centrally acting mechanism is the interference effect induced by PNS on long nociceptive fibers and pathways [38], specifically the medial lemniscal pathway, mediated by the inhibition of wide dynamic range neurons [39]. While all the mechanisms mentioned above likely contribute to the effect of PNS, the mechanisms targeted by high frequency and low intensity stimulation (inhibition of large fiber spinothalamic afferents), and low frequency and high intensity stimulation (activation of antinociceptive systems) [40] may explain how the utilization of a spectrum of frequency and intensity combinations, as seen in the studies included in this systematic review, all produce a positive pain reduction effect. 

Of the four studies included in this systematic review that demonstrated the ability of PNS to reduce neuropathic pain experienced by patients who suffered some form of traumatic nerve injury, three [22,25,26] of them recruited patients who underwent amputation procedures and one recruited patients with brachial plexus and upper extremity nerve injuries [21]. The authors query the utility of neuromodulation interventions to aid in the process of nerve regeneration and reinnervation following mechanical nerve injuries, which has recently gained more attention as evidence has emerged supporting this potential mechanism [41].

Building upon decades of preclinical research that demonstrated the ability of electrical stimulation to accelerate axonal regeneration proximal to the site of nerve injury, four RCTs (3 of which were double blinded) demonstrated that a single session of low frequency electrical stimulation perioperatively was able to improve the outcomes of patients suffering from ulnar [42] and median nerve [43] (increase in motor unit number estimation), digital nerve [44] (multimodal sensory function), and spinal accessory nerve [45] (functional outcomes) injuries. The promising findings of these studies provide an opportunity for the incorporation of brief, perioperative, low frequency electrical stimulation into the standard of care for surgical peripheral nerve injury management. The exciting prospect of a multimodal approach to peripheral nerve injury management combining electrical stimulation, end-to-end nerve autografts, and local administration of FK506 (Tacrolimus), a drug that has also proven the ability to accelerate nerve regeneration, to the site of coaptation in transected nerves may further improve patient outcomes in the future [46]. 

Strengths of this systematic review are the inclusion of only prospective studies, query of multiple databases, and appraisal of both bias risk and evidence quality [47]. However, the findings of our study should be interpreted while taking into account some notable limitations. A common study design of included studies was prospective case series, which lack generalizability. Three [25,26,27] of the included studies had a partial crossover design, hence introducing the possibility of carryover effect and the subsequent impact on the outcome. None of the three studies implemented a reasonable washout period [48]. The subjective nature of our primary and secondary outcomes in addition to challenges with choosing a specific study population for every study may introduce ambiguity in conclusions. Another limitation is that several studies included and analyzed only patients who showed improvement in their pain [16,17,26]. We did not assess opioid and non-opioid analgesic consumption in our review. The relatively short follow-up periods of included studies also add uncertainty regarding the long-term effects and potential adverse effects from PNS therapy. Variations in implantation sites, techniques, level of proceduralists’ expertise, and waveform settings are all confounding factors that may markedly impact the results. This degree of clinical and methodological heterogeneity also averted the possibility of performing a meta-analysis. There are numerous causes and inducers of peripheral neuropathy that were not accounted in this systematic review including traumatic neurovascular injuries, infections (e.g., human immunodeficiency virus infection), inflammatory causes (e.g., Guillain-Barre syndrome), hereditary disorders (e.g., Charcot-Marie-Tooth disease), and systemic disease (e.g., diabetes mellitus). Even rare causes such as pelvic malignancy and other pelvic pathologies may induce peripheral neuropathy [49,50].

PNS utilization in standard clinical practice is increasing as evidence continues to grow, supporting its mechanism of action, safety profile and clinical efficacy. Future advancements in device technology, surgical technique, waveform delivery and electrical programming will likely open the possibility of neural target optimization, allowing better understanding of responders to this therapy, thereby improving patient selection, and optimizing longitudinal efficacy and safety. For future directions, PNS indications may expand from targeting only localized neuropathic pain to more diffuse and complex painful syndromes. Although additional research is needed, this emerging therapy may have the potential to significantly change practice patterns and could substantially impact patient satisfaction and quality of life in patients suffering from intractable chronic neuropathic pain. Finally, loss of efficacy from neuromodulation interventions has been described and strategies to salvage efficacy from implanted neuromodulation devices warrant future investigation [51].

## 5. Conclusions

This review highlighted low-quality GRADE evidence supporting the use of PNS therapy to treat peripheral neuropathic pain. Further, studies highlight promising data on improvement in neurological function, quality of life, satisfaction, and emotional functioning after PNS therapy for peripheral neuropathic pain.

## Figures and Tables

**Table 1:** Summary of Studies.

Author/Year	Study Design	Study Funding Source	Mean Age of Subjects	Type of Interventions	Waveform Settings	Sample Size	Follow-Up Period	Primary Outcome	Secondary Outcomes
Frederico 2020 [14]	Prospective longitudinal case series	No funding	42.2	7 days PNS trial. Patients with >50% improvement in pain had permanent PNS implants.	Pulse width = 210 μs, freq = 40–60 Hz, and amplitude from 0.6 mA to 1.7 mA.	14 (10 were included)	12 months	8/10 patients (80%) have >50 pain reduction and 2/10 have 30% pain reduction on VAS scale. 60.2% improvement in neuropathic pain.	21.9% improvement in quality of life.
Glimore 2020 (12 Month Follow up for Gilmore 2019) [25]	Multicenter, randomized, double-blind, placebo-controlled, partial-crossover study	Industry and non-industry funding	46.5	Temporary PNS	Asymmetric charge-balanced biphasic pulse train. Pulse width = 10–200 μs, freq = 100 Hz, and amplitude 1–30 mA	47 (26 were included ana analyzed for efficacy)	12 months	67% of participants in group 1 (i.e., treatment group) had >50% reductions in pain in all qualifying regions of RLP and PLP. 0% of group 2 (i.e., placebo group) reported ≥50% reductions in average weekly pain at the end of the placebo period.	56% of participants in group 1 (i.e., treatment group) reported ≥50% reductions in pain interference in all qualifying regions of RLP and PLP at the end of the 12-month follow-up, compared with 18% in group 2 at the end of the placebo period. Reduction in pain interference with general activity, walking, sleeping, enjoyment of life by 55%, 39%, 63% and 65%, respectively. For treatment group, average BDI-II score was 55% lower than baseline at the end of 8 weeks of PNS and remained 33% lower than baseline at 12 months.
Glimore 2019 [26]	Multicenter, randomized, double-blind, placebo-controlled, partial-crossover study	Industry and non-industry funding	46.5	Temporary PNS	Asymmetric charge-balanced biphasic pulse train. Pulse width = 10–200 μs, freq = 100 Hz, and amplitude 1–30 mA	47 (26 were included ana analyzed for efficacy)	12 months	7/12 (58%) of patients receiving PNS reported >50% pain relief compared to only 2/14 (14%) in placebo group during weeks 1–4 of therapy. Among these 7 patients’ average reductions in RLP and PLP were 73% and 69%, respectively. 8/12 (687%) of patients receiving PNS reported >50% pain relief compared to only 2/14 (14%) in placebo group during weeks 5–8 of therapy. Among these 7 patients’ average reductions in RLP and PLP were 56% and 72%, respectively. After crossing over at week 4, patients in the placebo group reported only significant improvement in PLP but not RLP, subjects reporting >50% pain improvement remained 2/14 (14%).	8/10 (80%) of patients receiving PNS reported >50% reductions in average pain interference in all qualifying regions of RLP and PLP at the end of the treatment period compared to 2/13 (15%) in placebo group. PGIC score was 2.2 in PNS group compared to 0.6 in placebo group. After crossing over at week 4, PGIC increased to 1.3 in placebo group.
Oswold 2019 [15]	Prospective case series	Industry funding	N/A	Permanent PNS	Phase duration: 70–500 ms, freq = 0–200 Hz and amplitude of 1–30 mA	39 patients (42 PNS implants	6 months	78% of patients had improvement in their pain, with an average of 71% reduction. Average VAS pain score decreased from 8 cm pre procedural to 2 cm post-implants. Greatest reduction in pain scores with lateral femoral cutaneous nerve (100% reduction). Smallest pain score improvement (29%) with the intercostal nerve stimulation.	100% of patients reported improvement in their physical activity with an average improvement of 72%. Greatest noted with the brachial plexus (80%) and suprascapular nerve (80%) and smallest in the intercostal nerve (40%).
Wilson 2018 [16]	Case series	Industry and non-industry funding	62.7	Temporary PNS	Pulse width ranges from 40–200 ms, freq = 12 Hz, and amplitude of 20 mA.	28 (5 underwent permanent implantation and were analyzed for efficacy)	24 months	100% of patients have pain reduction > 50% at 6 and 12 month follow up and about 80% had pain improvement at 24 months follow up.	Improvement in pain interference with ADL measured by BPI-SF9 by 93.5%, 95.9% and 91.1% at 6, 12 and 24 months, respectively, compared to end of sham period. Improvement in pain during shoulder external ROM by 46.2%, 56.7% at 6, 12 months, respectively, compared to end of sham period. Global impression of change by the patients were more towards much improvement.
Freitas 2017 [24]	Prospective longitudinal case series	No funding	32	7 days trial. Patients with >50% improvement in pain had permanent implants.	Low frequency tonic stimulation (Pulse Width = 180 msec, freq = 40 to 60 Hz and Amplitude from 0.5 to 2 mA	23 (10 underwent permanent implantation and were analyzed for efficacy)	12 months	60% of patients who underwent permanent device implantation showed a pain reduction of 50% or greater (75% reduction on average), and 20% showed a 30% reduction in pain.	There was an improvement in quality of life and a return to engagement in the activities of daily life (no % reported).
Sokal 2017 [25]	Prospective clinical trial study	No funding	59.3	Permanent PNS	Intermittent stimulation with a pulse width of up to 800 μs, freq up to 40 Hz, and amplitude of up to 18 mA.	6	6 months	Average VAS score 2.6, 1.6 and 1.3 at 1, 3 and 6 months, respectively, down from 7.5 at baseline. Average short-form McGill pain questionnaire score was 11, 6.3 and 4.5 at 1, 3 and 6 months, respectively, down from 23.8 at baseline.	N/A
Deer 2016 [26]	Prospective, Multicenter, Randomized, Double-Blinded, Partial Crossover Study	Industry funding	53	Permanent PNS	Pulse width = 200 μs; Freq = 100 Hz, with amplitude set for paresthesia.	147 (94 underwent implantation and were analyzed for efficacy)	3 months for efficacy and 1 year for safety	27% reduction in pain in treatment group compared to 2.3% reduction in control group at 3 months follow up. Treatment group had significant improvement in worst pain score.	Treatment group had significant improvement in BPI score for general activity, mood, walking, normal work, relations to other people, sleep, and enjoyment in life, overall quality of life related to the painful condition and better global impression of degree of satisfaction.
Voorbrood 2015 [27]	Prospective study	Industry funding	53	Permanent PNS	N/A	37 (7 patients received PNS)	3 months	Reduction of pain on the NRS scale from 8 to 2.	N/A
Wilson 2014 [28]	Case series	Industry and non-industry funding	52.2	Temporary PNS	Pulse width range of 20–200 μs, freq 12 Hz, and amplitude of 20 V.	10	3 months	36.6% reduction in pain at end of treatment, 35.4% reduction at 5-week follow up, 40.2% reduction at 8-week follow up, and 48.8% reduction at 16-week follow up.	45.5% reduction in shoulder related disability at end of treatment (EOT), 37.4% reduction at 5-week follow up, 53.7% reduction at 8-week follow up, and 47.5% reduction at 16-week follow up. 52% reduction in pain Interference with ADL at EOT, 46% reduction at 5-week follow up, 60% reduction at 8-week follow up, and 58% reduction at 16-week follow up. 47.8% increase in range of Motion (ROM) at 8-week follow up, and 48.6% increase at 16-week follow up. Improvement in quality of Quality of life (PGIC scale) for 8/10 patients (80%) at EOT and 5/8 (62.5%) at week 16
Stevanato 2014 [29]	Open label trial	No funding	46	Permanent PNS	Pulse width = 250, freq = 50 Hz, and amplitude ranging from 0.15 to 0.30 V	7	12 months	Reduction of NRS pain score from 9 before surgery to 2.14 at the 6-month follow-up and to 2.57 at the 12-month follow-up.	N/A
Rauck 2014 [30]	Case series	No funding	47	Temporary PNS	Pulse width = 10–40 μs, freq 50–100 Hz, and amplitude of 1–20 mA	16 (9 analyzed for efficacy)	1 month	56% reduction in the mean of worst daily post-amputation pain in the second week and forth week of stimulation. 8/9 patients (89%) reported clinically significant relief during the second week of stimulation, and 7/9 (78%) reported significant relief during the fourth week of follow-up. Significant decrease in average pain, pain interference and pain disability Index (PDI) scores in the second week and fourth week of follow up.	Small non-significant decreases in depression scores (BDI-II). Improvement in quality of life with the assessment of the patient global impression of change in the second week and fourth week of follow up.
Deer 2010 [31]	Single-center open-label prospective feasibility trial	No funding	53.7	Temporary PNS	Pulse width (100 to 300 ms), freq 20 to 45 Hz). amplitude (<80 mA)	8 patients (10 implants, i.e., each implant was considered a separate patient)	1–2 weeks	2/10 patients (20%) have a >30% decrease in pain. Reduction of mean average pain score pain to 6.7 preimplant to 6.2 at the post explant follow-up visit V5. 9/10 (90%) experienced 17–100% reduction in pain intensity on day 5 (at the end of stimulation) versus baseline, with an average pain reduction of 44.2%. 1/10 (10%) experienced a 17% increase in pain intensity on day 5 (at the end of stimulation) versus baseline. N.B After explant, pain returned to baseline, increasing 36.8% to 45.6% relative to average reduced pain with daily stimulation.	Overall satisfaction score with the study was 9.6 cm, on a scale from 0 to 10 cm. All patients (100%) responded by selecting 10/10 (with 10 meaning ‘‘complete likelihood’’) as to their likelihood for wanting to undergo similar treatment with a permanent device.
Hassenbusch 1996 [32]	Prospective, consecutive series	No funding	N/A	Permanent PNS	N/A	32 (30 underwent permanent PNS placement were analyzed for efficacy)	2–4 years	19/30 patients (63%) experienced Long-term pain relief. 10/19 patients (52.6%) had good long-term relief and 9/19 (47.43%) had fair relief. Pain decreased from 8.3 preimplantation to 3.5 at the latest follow up on verbal digital scale. 60.9% reduction in allodynia.	Marked improvement in patient activity levels and vascular motor tone; however, less improvement in motor weakness and trophic changes. Activity levels increased by 63.3% in the success group between preimplantation and last follow-up evaluations. (i.e., success group are patients who experienced pain relief). 6/30 patients (20%) returned to part-time or full-time work after being unemployed before stimulator implantation.

ADL: activity of daily life; BDI: Beck’s depression inventory; EOT: end of treatment; Freq: Frequency; Hz: hertz; mA: milli ampere; ms: milli second; NIH: national institutes of health; NRS: Numerical rating scale; V: volt; VAS: visual acuity score; PGIC: patient global impression of change; PLP: phantom limb pain; RLP: residual limb pain; μs: micro second.

**Table 2:** Newcastle Ottawa risk assessment, table and paragraph.

Author	Year	Selection	Comparability	Outcome
Peripheral Nerve Stimulation Studies				
Frederico et al. [14]	2020	***	-	***
Oswold et al. [15]	2019	***	-	**
Freitas et al. [17]	2019	***	-	**
Sokal et al. [18]	2017	***	-	***
Wilson et al. [20]	2014	***	-	***
Stevanato et al. [21]	2014	***	-	***
Deer et al. [23]	2010	***	-	**
Hassenbusch et al. [24]	1996	***	-	**

* Quality of cohort and case–control studies was determined using the Newcastle-Ottawa scale, which evaluates three categories: selection (maximum four stars), comparability (maximum two stars), and outcome (maximum three stars).

**Table 3:** GRADE Assessment.

Certainty Assessment							Impact	Certainty
№ of Studies	Study Design	Risk of Bias	Inconsistency	Indirectness	Imprecision	Other Considerations		
**CRPS Pain**								
3	observational studies	serious ^a^	not serious	not serious	not serious	strong association	All 3 studies reported improvements in pain caused by CRPS with avergage reductions in pain scores ranging from 56% to 83%	⨁⨁◯◯ Low
**Shoulder Pain**								
2	observational studies	not serious	not serious	not serious	serious ^b^	strong association	Both studies reported improvements in pain, ranging from 48.8% to 80% reductions.	⨁⨁◯◯ Low
**Phantom Limb Pain**								
3	observational studies (2 RCTs)	not serious	not serious	not serious	not serious	strong association	All three studies reported reductions in pain. Average reductions were greater than 50%. In the RCT and its follow up, more patients in the PNS group experienced significant long term pain relief.	⨁⨁⨁◯ Moderate
**Post-Surgical Pain**								
2	observational studies (1 RCT)	not serious	not serious	not serious	not serious	none	Both studies reported improvement in pain. Average pain score reductions ranged from 27% to 75%. In one RCT, the PNS group had greater reductions in pain scores than the control (27% compared to 2.3%)	⨁⨁◯◯ Low
**Mononeuropathy Pain**								
5	observational studies	serious ^a^	not serious	not serious	not serious	strong association	All 5 studies reported improvements in pain caused by mononeuropathy. Average reductions in pain scores ranged from 36–71%	⨁⨁◯◯ Low

Explanations: ^a^. High level of heterogeneity within and across studies; moderate risk of outcome bias; ^b^. Low power due to only 15 patients being analyzed total between the two studies.

## Data Availability

Not applicable.

## Supplementary Materials

The following supporting information can be downloaded at: https://www.mdpi.com/article/10.3390/biomedicines10102606/s1; Table S1: Number of results per database. Table S2: Ovid Search Syntax. Table S3: Scopus Search Syntax.
