# Supplementary material for: Implantable Peripheral Nerve Stimulation for Peripheral Neuropathic Pain: A Systematic Review of Prospective Studies
Source: Biomedicines. 2022 Oct 17;10(10):2606. doi: 10.3390/biomedicines10102606 (PMC9599770; doi:10.3390/biomedicines10102606)
Supplement: Supplementary file 1 [file biomedicines-10-02606-s001.zip › biomedicines-1962675 supplementary.pdf]

Table S1. Number of results per database.

Database	Results
MEDLINE(R) and Epub Ahead of Print, In-Process, In-Data-Review & Other Non-Indexed Citations	547
Embase	633
Cochrane Central Register of Controlled Trials	144
Cochrane Database of Systemic Reviews	1
Scopus	55

Table S2. Ovid Search Syntax.

Searches	Results
1. exp Peripheral Nervous System Diseases/	246376
2. exp peripheral nerves/	293723
3. exp pain/	2007784
4. 2 and 3	25139
5. ((peripheral and nerve* and pain*) or Acrodynia or "Alcoholic Neuropath*" or "Alstrom syndrome*" or "amputation pain*" or "Amyloid Neuropath*" or "Brachial Plexus Neuritis" or "Brachial Plexus Neuropath*" or "Carpal Tunnel syndrome*" or "Cauda Equina syndrome*" or Causalgia or "Cervical Rib syndrome*" or "Charcot-Marie-Tooth Disease" or "Complex Regional Pain Syndrome*" or "Congenital Pain Insensitivit*" or "Cubital Tunnel syndrome*" or "Diabetic Neuropath*" or "Familial Dysautonomia*" or "Femoral Neuropath*" or "Giant Axonal Neuropath*" or "Guillain-Barre syndrome*" or "Hand-Arm Vibration syndrome*" or "Hereditary Sensory and Autonomic Neuropath*" or "Hereditary Sensory and Motor Neuropath*" or "Isaacs syndrome*" or "Median Neuropath*" or "Miller Fisher syndrome*" or Mononeuropath* or "Morton Neuroma*" or "Neonatal Brachial Plexus Pals*" or "Nerve Compression Syndrome*" or "Nerve Sheath Neoplasm*" or Neuralgia* or Neuritis or Neurofibroma* or "Neurofibromatosis 1" or neuropath* or "neuropathic pain" or "Paraneoplastic Polyneuropath*" or "peripheral nerve disease*" or "Peripheral Nerve Injur*" or "Peripheral Nervous System disease*" or "peripheral nervous system disorder*" or "Peripheral Nervous System Neoplasm*" or "peripheral neuropath*" or "Peroneal Neuropath*" or "phantom limb pain*" or "phantom pain*" or "Piriformis Muscle syndrome*" or "pns disease*" or "POEMS syndrome*" or Polyneuropath* or Polyradiculoneuropath* or Polyradiculopath* or "postamputation pain*" or "Pudendal Neuralgia" or "Radial Neuropath*" or Radiculopath* or "Reflex Sympathetic Dystroph*" or "Refsum Disease" or "Sciatic Neuropath*" or Sciatica or "Small Fiber Neuropath*" or "Tangier Disease" or "Tarlov Cyst*" or "Tarsal Tunnel	635015

	syndrome*" or "Thoracic Outlet syndrome*" or "Tibial Neuropath*" or "Ulnar Nerve Compression Syndromes" or "Ulnar Neuropath*").ti,ab,kf.	
6.	1 or 4 or 5	733737
7.	("peripheral nerve stimulation*" or "peripheral nerve stimulator*" or "PN stimulat*").ti,ab,kf.	4773
8.	6 and 7	2022
9.	limit 8 to english language [Limit not valid in CDSR; records were retained]	1953
10.	limit 8 to no language specified [Limit not valid in CDSR; records were retained]	8
11.	9 or 10	1960
12.	(exp animals/ or exp nonhuman/) not exp humans/	11871688
13.	((alpaca or alpacas or amphibian or amphibians or animal or animals or antelope or armadillo or armadillos or avian or baboon or baboons or beagle or beagles or bee or bees or bird or birds or bison or bovine or buffalo or buffaloes or buffalos or "c elegans" or "Caenorhabditis elegans" or camel or camels or canine or canines or carp or cats or cattle or chick or chicken or chickens or chicks or chimp or chimpanze or chimpanzees or chimps or cow or cows or "D melanogaster" or "dairy calf" or "dairy calves" or deer or dog or dogs or donkey or donkeys or drosophila or "Drosophila melanogaster" or duck or duckling or ducklings or ducks or equid or equids or equine or equines or feline or felines or ferret or ferrets or finch or finches or fish or flatworm or flatworms or fox or foxes or frog or frogs or "fruit flies" or "fruit fly" or "G mellonella" or "Galleria mellonella" or geese or gerbil or gerbils or goat or goats or goose or gorilla or gorillas or hamster or hamsters or hare or hares or heifer or heifers or horse or horses or insect or insects or jellyfish or kangaroo or kangaroos or kitten or kittens or lagomorph or lagomorphs or lamb or lambs or lemur or lemurs or llama or llamas or macaque or macaques or macaw or macaws or marmoset or marmosets or mice or minipig or minipigs or mink or minks or monkey or monkeys or mouse or mule or mules or nematode or nematodes or octopus or octopuses or orangutan or "orang-utan" or orangutans or "orang-utans" or ostrich or ostriches or oxen or parrot or parrots or pig or pigeon or pigeons or piglet or piglets or pigs or porcine or primate or primates or quail or rabbit or rabbits or rat or rats or reptile or reptiles or rodent or rodents or ruminant or ruminants or salmon or sheep or shrimp or slug or slugs or swine or tamarin or tamarins or toad or toads or trout or urchin or urchins or vole or voles or waxworm or waxworms or wildlife or worm or worms or xenopus or "zebra fish" or zebrafish) not (human or humans or patient or patients)).ti,ab,hw,kf.	10141571
14.	11 not (12 or 13)	1858

15. limit 14 to (conference abstract or editorial or erratum or note or addresses or autobiography or bibliography or biography or blogs or comment or dictionary or directory or interactive tutorial or interview or lectures or legal cases or legislation or news or newspaper article or overall or patient education handout or periodical index or portraits or published erratum or video-audio media or webcasts) [Limit not valid in CCTR,CDSR,Embase,Ovid MEDLINE(R),Ovid MEDLINE(R) Daily Update,Ovid MEDLINE(R) PubMed not MEDLINE,Ovid MEDLINE(R) In-Process,Ovid MEDLINE(R) Publisher; records were retained]	534
16. from 15 keep 1	1
17. (14 not 15) or 16	1325
18. remove duplicates from 17	780

Table S3. Scopus Search Syntax.

**Searches**
1. TITLE-ABS-KEY((peripheral and nerve* and pain*) OR Acrodynia OR "Alcoholic Neuropath*" OR "Alstrom syndrome*" OR "amputation pain*" OR "Amyloid Neuropath*" OR "Brachial Plexus Neuritis" OR "Brachial Plexus Neuropath*" OR "Carpal Tunnel syndrome*" OR "Cauda Equina syndrome*" OR Causalgia OR "Cervical Rib syndrome*" OR "Charcot-Marie-Tooth Disease" OR "Complex Regional Pain Syndrome*" OR "Congenital Pain Insensitivit*" OR "Cubital Tunnel syndrome*" OR "Diabetic Neuropath*" OR "Familial Dysautonomia*" OR "Femoral Neuropath*" OR "Giant Axonal Neuropath*" OR "Guillain-Barre syndrome*" OR "Hand-Arm Vibration syndrome*" OR "Hereditary Sensory and Autonomic Neuropath*" OR "Hereditary Sensory and Motor Neuropath*" OR "Isaacs syndrome*" OR "Median Neuropath*" OR "Miller Fisher syndrome*" OR Mononeuropath* OR "Morton Neuroma*" OR "Neonatal Brachial Plexus Pals*" OR "Nerve Compression Syndrome*" OR "Nerve Sheath Neoplasm*" OR Neuralgia* OR Neuritis OR Neurofibroma* OR "Neurofibromatosis 1" OR neuropath* OR "neuropathic pain" OR "Paraneoplastic Polyneuropath*" OR "peripheral nerve disease*" OR "Peripheral Nerve Injur*" OR "Peripheral Nervous System disease*" OR "peripheral nervous system disorder*" OR "Peripheral Nervous System Neoplasm*" OR "peripheral neuropath*" OR "Peroneal Neuropath*" OR "phantom limb pain*" OR "phantom pain*" OR "Piriformis Muscle syndrome*" OR "pns disease*" OR "POEMS syndrome*" OR Polyneuropath* OR Polyradiculoneuropath* OR Polyradiculopath* OR "postamputation pain*" OR "Pudendal Neuralgia" OR "Radial Neuropath*" OR Radiculopath* OR "Reflex Sympathetic Dystroph*" OR "Refsum Disease" OR "Sciatic Neuropath*" OR Sciatica OR "Small Fiber Neuropath*" OR "Tangier Disease" OR "Tarlov Cyst*" OR "Tarsal Tunnel syndrome*" OR "Thoracic Outlet syndrome*" OR "Tibial Neuropath*" OR "Ulnar Nerve Compression Syndromes" OR "Ulnar Neuropath*")
2. TITLE-ABS-KEY("peripheral nerve stimulation*" OR "peripheral nerve stimulator*" OR "PN stimulat*")
3. LANGUAGE(english)

4.	1 and 2 and 3
5.	TITLE-ABS-KEY((alpaca OR alpacas OR amphibian OR amphibians OR animal OR animals OR antelope OR armadillo OR armadillos OR avian OR baboon OR baboons OR beagle OR beagles OR bee OR bees OR bird OR birds OR bison OR bovine OR buffalo OR buffaloes OR buffalos OR "c elegans" OR "Caenorhabditis elegans" OR camel OR camels OR canine OR canines OR carp OR cats OR cattle OR chick OR chicken OR chickens OR chicks OR chimp OR chimpanze OR chimpanzees OR chimps OR cow OR cows OR "D melanogaster" OR "dairy calf" OR "dairy calves" OR deer OR dog OR dogs OR donkey OR donkeys OR drosophila OR "Drosophila melanogaster" OR duck OR duckling OR ducklings OR ducks OR equid OR equids OR equine OR equines OR feline OR felines OR ferret OR ferrets OR finch OR finches OR fish OR flatworm OR flatworms OR fox OR foxes OR frog OR frogs OR "fruit flies" OR "fruit fly" OR "G mellonella" OR "Galleria mellonella" OR geese OR gerbil OR gerbils OR goat OR goats OR goose OR gorilla OR gorillas OR hamster OR hamsters OR hare OR hares OR heifer OR heifers OR horse OR horses OR insect OR insects OR jellyfish OR kangaroo OR kangaroos OR kitten OR kittens OR lagomorph OR lagomorphs OR lamb OR lambs OR llama OR llamas OR macaque OR macaques OR macaw OR macaws OR marmoset OR marmosets OR mice OR minipig OR minipigs OR mink OR minks OR monkey OR monkeys OR mouse OR mule OR mules OR nematode OR nematodes OR octopus OR octopuses OR orangutan OR "orang-utan" OR orangutans OR "orang-utans" OR oxen OR parrot OR parrots OR pig OR pigeon OR pigeons OR piglet OR piglets OR pigs OR porcine OR primate OR primates OR quail OR rabbit OR rabbits OR rat OR rats OR reptile OR reptiles OR rodent OR rodents OR ruminant OR ruminants OR salmon OR sheep OR shrimp OR slug OR slugs OR swine OR tamarin OR tamarins OR toad OR toads OR trout OR urchin OR urchins OR vole OR voles OR waxworm OR waxworms OR worm OR worms OR xenopus OR "zebra fish" OR zebrafish) AND NOT (human OR humans or patient or patients))
6.	4 and not 5
7.	DOCTYPE(ab) OR DOCTYPE(ed) OR DOCTYPE(bk) OR DOCTYPE(er) OR DOCTYPE(no) OR DOCTYPE(sh)
8.	6 and not 7
9.	INDEX(embase) OR INDEX(medline) OR PMID(0* OR 1* OR 2* OR 3* OR 4* OR 5* OR 6* OR 7* OR 8* OR 9*)
10.	8 and not 9
